# Decoupling Actions from Consequences: Dorsal Hippocampal Lesions Facilitate Instrumental Performance, but Impair Behavioral Flexibility in Rats

**DOI:** 10.3389/fnbeh.2016.00118

**Published:** 2016-06-08

**Authors:** Sebastian Busse, Rainer K. W. Schwarting

**Affiliations:** Behavioral Neuroscience, Experimental and Biological Psychology, Philipps-University of MarburgMarburg, Germany

**Keywords:** sequential learning, instrumental behavior, action/outcome, excitotoxic lesions, dorsal hippocampus, SRTT, declarative memory, rat

## Abstract

The present study is part of a series of experiments, where we analyze why and how damage of the rat’s dorsal hippocampus (dHC) can enhance performance in a sequential reaction time task (SRTT). In this task, sequences of distinct visual stimulus presentations are food-rewarded in a fixed-ratio-13-schedule. Our previous study (Busse and Schwarting, 2016) had shown that rats with lesions of the dHC show substantially shorter session times and post-reinforcement pauses (PRPs) than controls, which allows for more practice when daily training is kept constant. Since sequential behavior is based on instrumental performance, a sequential benefit might be secondary to that. In order to test this hypothesis in the present study, we performed two experiments, where pseudorandom rather than sequential stimulus presentation was used in rats with excitotoxic dorsal hippocampal lesions. Again, we found enhanced performance in the lesion-group in terms of shorter session times and PRPs. During the sessions we found that the lesion-group spent less time with non-instrumental behavior (i.e., grooming, sniffing, and rearing) after prolonged instrumental training. Also, such rats showed moderate evidence for an extinction impairment under devalued food reward conditions and significant deficits in a response-outcome (R-O)-discrimination task in comparison to a control-group. These findings suggest that facilitatory effects on instrumental performance after dorsal hippocampal lesions may be primarily a result of complex behavioral changes, i.e., reductions of behavioral flexibility and/or alterations in motivation, which then result in enhanced instrumental learning.

## Introduction

It is our current understanding that memory can be divided into two main categories: declarative (memory of “what”) and non-declarative memory (memory of “how”). Further division leads to distinct sub-categories of memory, e.g., procedural memory, which is a certain type of non-declarative memory. This psychological classification is paralleled by a neurobiological one, since declarative memory is linked to structures like the hippocampus, whereas procedural memory is linked, among others, to basal ganglia function (McDonald and White, 1994). Both types of memory also require and partly share neocortical mechanisms. The scientific investigation of procedural learning and memory is based on specific tests, and one of the classical human ones is the serial (or sequential) reaction time task (SRTT; Nissen and Bullemer, 1987). In order to provide a translation of this task for rodents, we developed a SRTT in rats, where sequential instrumental nose-poking is reinforced under fixed-ratio conditions by food pellets (Domenger and Schwarting, 2006, 2007; for review, see Schwarting, 2009), using series of sequential stimuli which are identical to those used in typical research with humans (Nissen and Bullemer, 1987; Schwarting, 2009). Similar to what is known from patients with Parkinson’s disease (Ferraro et al., 1993; Nagy et al., 2007; Carbon et al., 2010), our early studies had shown that striatal dopamine lesions led to behavioral deficits (Domenger and Schwarting, 2008; Eckart et al., 2010). In contrast, and rather surprisingly, lesions of the dorsal hippocampus (dHC) led to substantial improvements, that is, they actually boosted performance in the SRTT (Eckart et al., 2012; Will et al., 2013; Busse and Schwarting, 2016). Thus, in our first dHC lesion study, where we analyzed sequential learning and performance, we found that rats with excitotoxic dHC lesions showed clearly faster reaction times (RTs) and higher response accuracy (ACC) as compared to controls. The same animals had the expected deficits in an object-place recognition task. That is, they were impaired in a so-called episodic memory task, which is usually attributed to hippocampal mechanisms, but showed improved performance in a procedural task thought to reflect striatal function. Similar, but less procedural improvements were obtained in a subsequent study (Will et al., 2013), where hippocampal neuron loss was restricted to its CA3 and CA1 regions. This was achieved by a specific method of perforant pathway stimulation, a paradigm that produces hippocampal granule cell discharges over a prolonged period of subsequent electrical stimulation (Norwood et al., 2010).

We discussed the mechanisms underlying our earlier findings in terms of the multiple parallel memory hypothesis (White and McDonald, 2002), which postulates competition or even interference in information processing between striatum and HC (for review, see White et al., 2013). Neuroanatomical studies show that both structures receive inputs from various cortices and project back into the prefrontal cortex (Coutureau and Killcross, 2003; Killcross and Coutureau, 2003; McDonald et al., 2007; Tait and Brown, 2007; Gruber and McDonald, 2012), where their simultaneous inputs may interfere depending on the demands of the given task. In an instrumental learning task like the SRTT, where the need for spatial information processing is greatly reduced due to the rather limited spatial arrangement of stimuli and reward delivery, loss of hippocampal interference may therefore facilitate required procedural learning processes.

Furthermore, lesion-induced loss of a direct or indirect hippocampal influence on the striatum is possible, since there are extensive connections between these structures (Groenewegen et al., 1987; McGeorge and Faull, 1989). However, apart from anatomical data, evidence for such influence is sparse and inconclusive. To demonstrate such an influence on a behavioral level would require an analysis of goal-directed and habitual behavioral systems: prolonged training results in a transfer of behavioral control from goal-directed to stimulus-response (S-R) habit systems (Yin and Knowlton, 2006) and the hippocampal formation may influence this process. If this would be the case, performance changes, especially improvements, in tasks that require primarily procedural information processing (such as the SRTT) would be expected following lesions of the HC.

In our early studies (Eckart et al., 2012; Will et al., 2013), where we found such improvements, the daily duration of training was kept constant. This factor may have favored learning and performance in rats with dHC lesions, which, due to their faster performance, obtained more practice each day. In order to rule this factor out, we performed another study (Busse and Schwarting, 2016), where daily training was ended whenever the rats had achieved a fixed number of successful instrumental responses and thus rewards. Again, rats with dHC lesions had deficits in an object-place recognition task, showing their typical declarative deficits. In the SRTT, however, they showed shorter RTs than controls only during initial SRTT-training. Nevertheless, they completed their daily trials faster than controls, and this result was largely due to the fact that the lesion-group showed shorter post-reinforcement pauses (PRPs). Also, they had impaired extinction behavior in a subsequent extinction test where reinforcement was withheld.

These findings led us to assume that the performance improvement of rats with dHC lesions in the SRTT might not specifically be due to an effect on sequential behavior, but to an effect on instrumental behavior, which is underlying our task. In order to test this hypothesis, we performed the present study where dHC lesions were applied as before; however, the rats were now trained under conditions of pseudorandom, rather than sequential stimulus presentation. Also, we asked whether the performance improvement (i.e., shorter daily session time and reduced PRPs), which we had observed in our prior studies, could have been the result of a reduction of behaviors that are not relevant to the task. Therefore, we monitored various operant and non-operant behaviors over the course of SRTT-training. Additionally, we took into account the fact that disruption of structures within the above mentioned memory systems can result in an overall deficit in behavioral flexibility. This deficit can have either adverse or even beneficial effects depending on the given task (Wirth et al., 1998; Cheung and Cardinal, 2005; Ito et al., 2005). Based on our recent findings, we assumed that damage to the dHC might result in a faster transition of goal-directed behavior into habitual behavior, which could explain the observed procedural performance facilitation, as well as the changes in behavior during the instrumental task. If this would be the case, dHC-ablated rats should show deficits in behavioral flexibility, as well as lesser awareness of response-outcome (R-O)-relationships. To analyze these facets, we examined extinction behavior under devalued reward-conditions, and behavioral flexibility when confronting rats with a R-O-dissociated version of the SRTT.

## Materials and Methods

### Animals and Maintenance

Forty-one male Wistar rats (Harlan Laboratories GmbH, Netherlands), weighing 250–274 g on the day of surgery, were used and they served in two distinct experiments (Experiment 1: *n* = 25; Experiment 2: *n* = 16). Animals were housed individually and had *ad libitum* access to water (room temperature: 21–25°C; 31–47% humidity; 12:12 h light/dark cycle). Prior to surgery, the animals were handled for three consecutive days (5 min each per day). The experiments were conducted in accordance with the ethical regulations for animal experimentation at the Philipps-University of Marburg and were approved by the German animal welfare authorities (Regierungspräsidium Gießen).

### Surgery

Surgery was performed similarly to our previous study: animals were anesthetized with isoflurane (Baxter Deutschland GmbH, Germany) and coordinates from bregma, as well as injection volumes for dHC lesion were identical to our previous study (for details, see Busse and Schwarting, 2016). Ibotenic acid and saline injections were made using a home-made injection system with a 1 μl SGE syringe (SGE Analytical Science), that was connected via polyethylene tubing (0.38 mm × 1.09 mm diameter; Plastics One Inc., VA, USA) to the injection cannula (gauge 26, Plastics One Inc., VA, USA). In order to allow the ibotenic acid or saline to diffuse, the syringe was kept in place for about 1–2 min after each injection (Busse and Schwarting, 2016).

#### Experiment 1

The lesion-group (*n* = 11) was injected with ibotenic acid (10 mg/ml in 0.1 M phosphate-buffered saline; RandD Systems GmbH, Germany). For sham-surgery (*n* = 6) only phosphate-buffered saline was injected and control animals (*n* = 8) underwent no surgical treatment.

#### Experiment 2

Only two groups were used (lesion-group: *n* = 9; control-group: *n* = 7), which underwent the same procedures as the respective groups in Experiment 1.

In both experiments, 14 days of recovery time were given after the surgery.

### SRTT

During SRTT-shaping and -training, the animals received food only during (food pellets, see below) and directly after daily instrumental sessions (Altomin rat chow; Altrumin, Germany). After each session, they were fed individually (according to their body weight) with weighted portions of Altromin chow, which assured that all rats maintained 80–85% of their free feeding weight.

For SRTT-training and testing (for details, see Domenger and Schwarting, 2008; Eckart et al., 2012), we used modified operant chambers (MedAssociates Inc., UK) with four LED-equipped holes (i.e., nose-poke holes) arranged in a small recess in a semi-elliptic way tilted towards the pellet-receptacle (photographs of the set-up can be found in Schwarting, 2009). The holes were numbered as follows: (1) upper left; (2) upper right; (3) bottom left; (4) bottom right. The pellet-receptacle was connected to a dispenser, which delivered adjustable pellet amounts (dustless precision pellets, 45 mg each; Bioserve, Bilaney Consultants, Germany). During the training session, the animals had to respond to visual stimuli by poking into the illuminated (i.e., active) hole. After a correct response, the light was immediately lit in another hole. The order of the illuminated holes was either pseudorandom (i.e., holes were illuminated randomly, but the same hole was never lit twice in a row) or followed a 12-item-sequence (3-2-4-1-3-4-2-1-2-3-1-4; second-order conditional sequence, for details, see Reed and Johnson, 1994). In order to ensure dissociation of sequence and reinforcement, the food reward was delivered after each 13th correct response (fixed ratio schedule of 13; FR-13).

#### Shaping

Similar to our previous studies (Eckart et al., 2012; Busse and Schwarting, 2016), the animals were trained daily for 20 min each during a 6–7 day long phase until they reached the criterion of FR-13. After an incorrect poke (i.e., a poke into one of the non-lit holes) two discriminative stimuli were presented: a bright light (house light) and a high-pitched tone (duration: 2 s). After the criterion of FR-13 was reached, a 5 s time limit for poking was applied (termed omission).

#### SRTT-Analysis

All pokes into any non-lit hole were termed “incorrect pokes” (exception: two pokes into the same nose-poke hole within 1 s), while all pokes into the lit hole within the 5 s time limit were termed “correct pokes”. Response ACC was derived from the percentage of correct pokes from the number of total pokes. For RT analysis, only correct pokes on FR positions 2–13 were used. RT values on FR position 1 revealed the PRP, which describes the period between reward delivery and the following first nose-poke of the next FR-run. The time required to complete the 20 FR-13 runs per trial was termed session time (for details, see Busse and Schwarting, 2016).

#### Behavioral Recordings

All trials were recorded on video (cameras: “Nadelöhr Super Mini Kamera”, Abus, Germany; video capture device: “4 Kanal Digitalrekorder”, Abus, Germany) from two different angles (bird’s-eye view and view from the backside towards the food receptacle) inside the modified operant chambers. The recordings were analyzed visually by a trained observer who was blind to group assignments. Training days 1 and 15 of the pseudorandom stimulus presentation period were used to compare lesion- and control-group. Five different behavioral measures were taken: inactivity, grooming, rearing, sniffing, and operant behavior. (1) Inactivity was defined as the time span during which the animals were neither performing the task, nor showing any distinct movement or any other of the following behaviors. (2) Grooming periods consisted of respective movements directed to the face, snout, and/or torso. (3) Sniffing behavior was defined as a distinct movement of the snout and vibrissae lasting for at least 2 s. (4) Rearing behavior was characterized by the rats standing up on the two hind limbs either on- or off-wall (including the operant chamber wall). (5) Operant behavior was defined as the time period in which the rats were performing the given task. The time periods of all distinct behaviors were quantified in total [s].

### Specific Testing

#### Experiment 1

##### SRTT-training

Using the FR-13 schedule with pseudorandom stimulus presentation, the animals were trained daily for an amount of 20 complete FR-13 runs, i.e., the number of reinforced runs was identical between subjects, whereas depending on speed and/or ACC of each subject, the test duration differed. Training proceeded until all groups showed stable levels of performance in terms of no further significant changes in RT, ACC, or session time over three consecutive days. This level was reached after 17 days of training.

##### Random-sequence test

On day 17, stimulus presentation was switched to the sequential 12-item-sequence for 6 more days of training in order to find possible performance improvements that usually occur under sequential conditions.

##### Devaluation-extinction-test

On day 23, the devaluation-extinction-test was conducted. Here, the previous sequential 12-item-sequence with an FR-13 schedule was used. Unlike during SRTT-training, the amount of FR-13 runs to complete was not limited. During the trial, the food dispenser was disconnected, so that it could not produce acoustic cues, which it normally produces after each 13th correct poke. Furthermore, no omission was used and a discriminative stimulus (house-light/tone) was only given after an incorrect poke. A non-devalued extinction-test in our prior study revealed significant impairments in extinction behavior after dHC lesion (Busse and Schwarting, 2016). In order to rule out that this impairment was caused by differences in the hedonic outcome of the food reward, we satiated all animals before the extinction-test in the present study. On the day before the test, all animals received 50 g of dustless precision pellets in addition to their normal amount of food pellets. This food remained in the cages until the test was conducted on the following day, which provided all animals with unrestricted access to food prior to extinction-testing (see Rossi and Yin, 2012). Animals were weighed every day and a significant weight gain on the day of the extinction-test in comparison to the day before confirmed that the pre-feeding was successful (mean weight gain = 30.64 ± 1.18 g; repeated measures ANOVA effect factor days: *F*_(1,22)_ = 616.085; *p* < 0.001; no group difference was found). Our previous study (Busse and Schwarting, 2016) also showed that animals would not abruptly cease poking when no food reward was delivered, but rather gradually reduce performance and show fewer responses, as well as longer pauses between pokes over time. In order to factor in this behavioral pattern, the program ended automatically if an animal had ceased poking for at least 3 min (cut-off), whereas any nose-poke (wrong or correct) before the cut-off would reset this timer. The total time until the cut-off (session time) and the amount of completed sequences were analyzed and compared between groups.

#### Experiment 2

##### SRTT-training

The training period was identical to that of Experiment 1, with the exception that only pseudorandom sequences were presented. A stable level of performance in terms of no further significant changes in RT, ACC, or session time over three consecutive days was reached after 18 days of training.

##### Response-Outcome-Dissociation

After completion of the SRTT-training period, the animals were tested again under the same pseudorandom 12-item-sequence with an FR-13 schedule. However, when eight full sequences were completed, the food reward was decoupled from the actions of the animal. Instead of delivering the food pellets after 13 correct pokes, the food reward was given automatically every 31.5 s and independent of the rat’s activities. Starting 1.5 s after food delivery, the 12-item-sequence was started and the animal could poke freely into each hole as before for a period of 30 s until the next food reward was given. Immediately after eight more food rewards (i.e., 252 s) had been ejected into the receptacle (16th reward overall), the program switched back to the normal FR-13 schedule where nose-pokes and food reward were coupled, that is, rewards depended on the animals actions again. After eight more completed sequences, the program switched a second time to a phase in which actions and food reward were decoupled (24th reward overall). After the 32th ejection of food pellets into the receptacle, the program stopped automatically. In summary, this program consisted of four phases: C1 (R-O-[c]oupled), D1 (R-O-[d]ecoupled), C2 (R-O-[c]oupled), and D2 (R-O-[d]ecoupled). All phases ended after the 8th reward, but the length of each C-phase was variable since it depended on the animal’s actions, while each D-phase lasted for 252 s. In total, 32 food rewards were given per training session. This test was repeated on 10 consecutive days. Since the durations of both C-phases were variable, whereas those of both D-phases were fixed, we calculated the amount of pokes per second (PPS; correct and incorrect pokes) to provide a comparable measure of performance.

### Histological Analysis

At the end of both experiments, the rats were deeply anesthetized with sodium-pentobarbital (Release 300 mg/ml, WDT, Germany) and perfused transcardially with 0.9% saline and 4% paraformaldehyde in 0.1 M phosphate buffer, pH 7.4. Brains were removed, post-fixed and cryo-protected (4% paraformaldehyde—30% sucrose solution, homemade). Coronal sections of 50 μm were cut on a cryostat (Leica Mikrosysteme Vertrieb GmbH, Germany). Every fourth slice was mounted on a glass slide and stained with cresylviolet. Planimetric measurement was used in order to quantify the cross-sectional area of the HC for all 22 brains using an Axio Imager.M2 microscope (Zeiss, Germany) with a magnification of 2.5× (Eckart et al., 2012; Busse and Schwarting, 2016).

For each brain, the area (μm^2^) of the remaining intact hippocampal tissue (CA1–3 fields, dentate gyrus; excluding the subiculum) in both hemispheres was traced manually with Stereo Investigator 9 (MicroBrightField Inc., VT, USA). Area values for each hemisphere were summed and then multiplied with slice thickness, which resulted in the estimated hippocampal tissue volumes (mm^3^). For analysis, estimated volumes from control- and sham-group were defined as baseline (100%) and compared with the respective lesion-group (Busse and Schwarting, 2016).

### Statistical Analysis

SPSS (Version 21.0) was used for all statistical tests, while statistical power analysis was conducted with G*Power (Version 3.1). Kolmogorov-Smirnov tests for all data revealed normal distribution; therefore, only parametric tests were used (ANOVAs and Tukey-corrected *post hoc* tests). Greenhouse-Geisser-correction was used when violations of sphericity were present. *P*-values were defined as follows: <0.05 significant; ≤0.1 trend; >0.1 no difference. All results are expressed as Mean ± SEM.

## Results

### Hippocampal Tissue Reduction

Analysis of the stained brain tissue revealed extensive damage of the dHC in all lesion-group animals. Additionally, dorsal subiculum and intermediate HC displayed minor damage, while damage of the neocortex was only visible near the injection tracts. Analysis of brain tissue from the sham-group (Experiment 1) revealed no gross damage of the HC. The observed tissue damages were comparable to our previous study (Busse and Schwarting, 2016).

The quantitative histological analysis of the 25 brains obtained at the end of Experiment 1 revealed significant hippocampal volume reductions (*F*_(2,22)_ = 275.077; *p* < 0.001) in the lesion-group as compared to controls (left: −57.06 ± 4.71%, right: −58.76 ± 6.7%). Reductions of hippocampal volume in sham-operated animals were small and did not differ significantly from controls (left: −13.64 ± 16.80%, right: −7.58 ± 11.79%).

The respective analysis of the 16 brains from Experiment 2 also revealed significant hippocampal volume reductions (*F*_(1,14)_ = 280.999; *p* < 0.001) in the lesion-group as compared to controls (left: −42.80 ± 2.14%, right: −52.81 ± 2.49%).

### Behavioral Results—Experiment 1

#### Pseudorandom Stimulus Presentation

**Figure 1:**
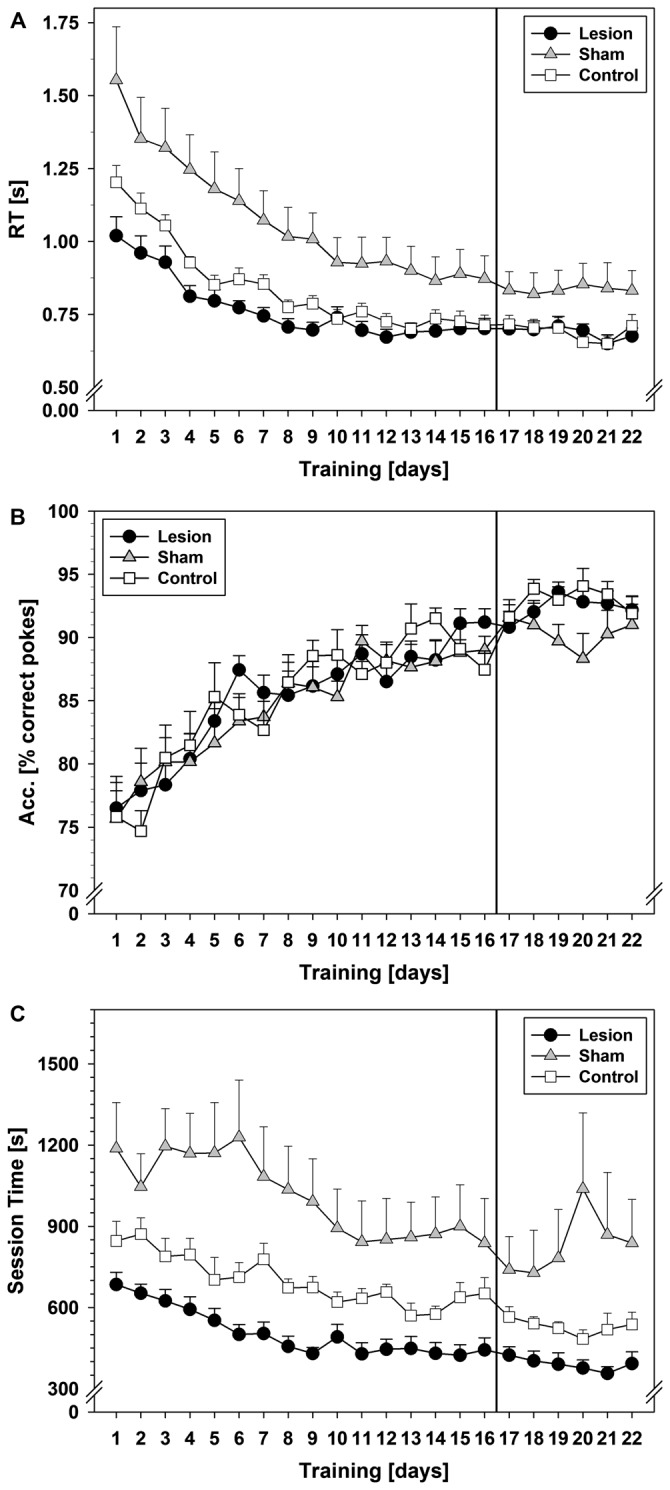
**SRTT data from Experiment 1.** The vertical line indicates the switch from pseudorandom to the subsequent sequential stimulus presentation. **(A)** Reaction times (RTs) in seconds during the 22-day training period. **(B)** Response accuracy (ACC) during the 22-day training period in terms of mean % of correct pokes. **(C)** Session times during the 22-day training period in seconds. Each data point reflects the mean of 20 successful fixed ratio schedule of 13 (FR–13) runs (±SEM). For statistical details see text.

Statistical analysis showed that RTs of the correct pokes (Figure 1A) decreased and became asymptotic in all groups (repeated measurement factor days: *F*_(3.624,79.731)_ = 94.971; *p* < 0.001). Further statistical tests revealed a significant group difference (*F*_(2,22)_ = 8.687; *p* = 0.002) and interaction between groups and training days (*F*_(7.248,79.731)_ = 4.126; *p* = 0.001). Subsequent *post hoc* tests showed longer RTs in the sham-group as compared to lesion (*p* = 0.001) or control (*p* = 0.02), which did not differ from each other.

ACC (Figure 1B) increased over the pseudorandom training period in all three groups (factor days: *F*_(5.868,129.105)_ = 26.715; *p* < 0.001) and reached ~90% correct pokes on training day 13. No significant group difference or interaction between training days and groups was found.

Session time (Figure 1C) decreased in all three groups (factor days *F*_(5.140,113.091)_ = 18.511; *p* < 0.001). A significant group effect (*F*_(2,22)_ = 13.042; *p* < 0.001) and an interaction between training days and groups (*F*_(10.281,113.091)_ = 2.031; *p* = 0.035) were found. *Post hoc* tests revealed longer session times in the sham-group as compared to control- (*p* = 0.019), or lesion-group (*p* < 0.001).

**Figure 2:**
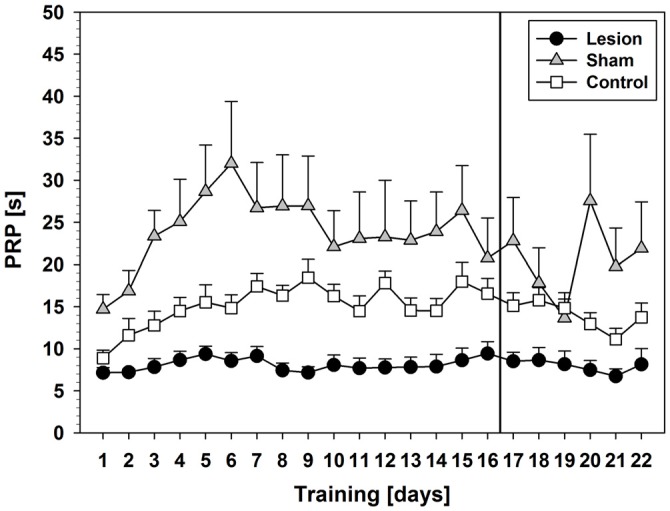
**Post-reinforcement pauses (PRPs) from Experiment 1 (Mean ± SEM).** The vertical line indicates the switch from pseudorandom to subsequent sequential stimulus presentation. For statistical details see text.

Comparison of PRPs (Figure 2) between groups during pseudorandom stimulus presentation showed a significant effect on factor training days (*F*_(5.375,118.254)_ = 8.231; *p* < 0.001) and an interaction between training days and groups (*F*_(10.750,118.254)_ = 2.978; *p* = 0.002). Furthermore, a significant group effect was found (*F*_(2,22)_ = 13.755; *p* < 0.001) and *post hoc* tests revealed significant shorter PRPs in the lesion-group as compared to sham (*p* < 0.001) or control (*p* = 0.048). Descriptively, control- and sham-group showed an increase in PRPs over the course of the first 6–9 training days and then remained on a stable level until day 16, while the lesion-group showed stable low levels of PRPs throughout the 16 days of training.

#### Sequential Stimulus Presentation

Starting on day 17, pseudorandom stimulus presentation was switched to sequential stimulus presentation (see black vertical line in Figures 1, 2). No statistical significant reduction in RT (Figure 1A) was found during this six-day training period, but similar to the previous pseudorandom phase, a significant group difference was revealed (*F*_(2,22)_ = 4.109; *p* = 0.03), that is, longer RTs in the sham-group as compared to the lesion- (*p* = 0.036) and a trend as compared to the control-group (*p* = 0.056).

No statistical differences were found in ACC during this sequential testing period. Also, no significant change (factor days) in session time was found, but the interaction between training days and groups showed a trend (*F*_(3.940,43.340)_ = 2.521; *p* = 0.056). Furthermore, a significant group difference was found (*F*_(2,22)_ = 7.843; *p* = 0.003). *Post hoc* tests revealed longer session times in the sham-group as compared to lesion-group (*p* = 0.002), but no statistical difference between lesion and control.

Comparison of PRPs between groups during sequential stimulus presentation showed a significant effect of the factor training days (*F*_(3.246,71.404)_ = 3.901; *p* = 0.010) and an interaction between training days and groups (*F*_(6.491,71.404)_ = 4.497; *p* < 0.001). Additionally, a significant group effect was found (*F*_(2,22)_ = 7.743; *p* = 0.003) and *post hoc* tests revealed significantly shorter PRPs in the lesion-group as compared to shams (*p* = 0.002), but not as compared to controls. Descriptively, control- and sham-group showed a decrease in PRP lengths, while the lesion-group still showed similar lengths of PRPs as during pseudorandom stimulus presentation.

#### Pseudorandom vs. Sequential Order

To test for possible differences between the two test phases, we computed means of the 6 days of sequential stimulus presentation and compared them with the respective means of the preceding 6 days of pseudorandom stimulus presentation (Table 1). This analysis yielded a significant difference in RTs between both phases (*F*_(2,22)_ = 14.432; *p* = 0.001), that is, shorter RTs during sequential stimulus presentation, and an interaction between phases and groups (*F*_(2,22)_ = 4.183; *p* = 0.029). A significant group difference was present (*F*_(2,22)_ = 5.011; *p* = 0.016), but *post hoc* testing revealed only smaller RTs in the lesion-group compared to shams (*p* = 0.016). Analysis of ACC showed an increase in correct pokes during sequential stimulus presentation (factor phases: *F*_(2,22)_ = 34.040; *p* < 0.001), but no interaction and no group difference. Session time decreased under sequential stimulus presentation (factor phases: *F*_(2,22)_ = 11.322; *p* = 0.003), but no interaction was found. However, a group difference was present (*F*_(2,22)_ = 8.868; *p* = 0.001) and *post hoc* testing revealed shorter session times in the lesion-group compared to shams (*p* = 0.001), but no difference between lesion- and control-group. PRPs decreased during sequential stimulus presentation (factor phases: *F*_(2,22)_ = 6.479; *p* = 0.018) and a group difference (*F*_(2,22)_ = 9.528; *p* = 0.001), but no interaction, was found. *Post hoc* analysis revealed shorter PRPs in the lesion-group compared to the sham-group (*p* = 0.001), but not between controls and lesion-group.

**Table 1:** **Comparison of pseudorandom and sequential stimulus presentation**.

	Pseudorandom stimulus presentation	Sequential stimulus presentation
**Response accuracy**
**(ACC in %)**
Lesion	89.04 ± 1.09	92.36 ± 0.76
Control	88.98 ± 1.04	92.97 ± 0.67
Sham	88.57 ± 1.13	90.31 ± 1.28
**Reaction time [s]**
Lesion	0.69 ± 0.03	0.69 ± 0.09
Control	0.73 ± 0.03	0.69 ± 0.08
Sham	0.90 ± 0.08	0.84 ± 0.17
**Session time [s]**
Lesion	436.81 ± 39.26	390.56 ± 33.12
Control	621.13 ± 35.49	528.18 ± 26.69
Sham	860.34 ± 137.70	832.68 ± 173.82
**Post-reinforcement pause [s]**
Lesion	8.21 ± 1.23	7.96 ± 1.28
Control	15.99 ± 1.29	13.93 ± 0.96
Sham	23.38 ± 4.97	20.58 ± 4.71

*Mean values of reaction time (RT), response accuracy (ACC), session time and post-reinforcement pauses (PRPs) from the last 6 days of pseudorandom stimulus presentation and the subsequent 6 days of sequential stimulus presentation are shown (Mean ± SEM)*.

#### Devaluation-Extinction-Test

**Figure 3:**
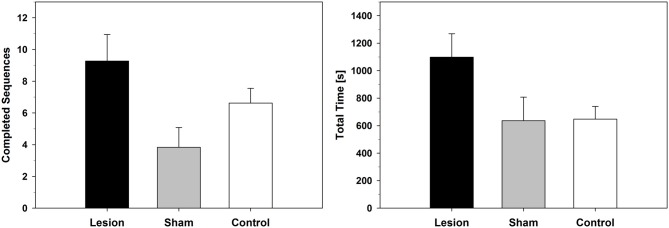
**Extinction-Test with devalued food reward.** Number of completed FR-13 runs (Mean ± SEM) and total time (Mean ± SEM) until a given animal stopped poking into any of the holes for at least 3 min. For statistical details see text.

Descriptively, the lesion-group completed more sequences and took longer until it reached the cut-off criterion, which was used as a measure of extinction (Figure 3). The statistical analysis, however, yielded only trends for differences between groups (extinction total time: *F*_(2,22)_ = 3.080; *p* = 0.066; completed sequences: *F*_(2,22)_ = 3.215; *p* = 0.060).

#### Behavioral Recordings and Analysis

This analysis was performed in order to get a better insight why session times and PRPs were substantially shorter in the lesion-group. We focused on the lesion-group in comparison to the control-group and summarized sniffing, grooming and rearing behavior under the category “non-operant behavior” for a more conclusive view.

**Figure 4:**
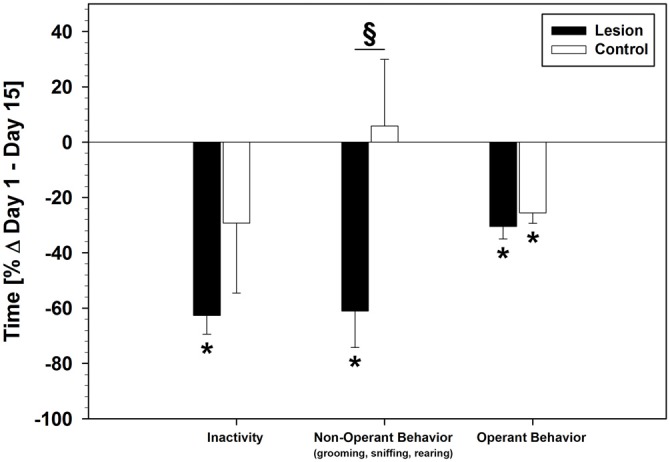
**Behavioral changes during the SRTT-training period under pseudorandom stimulus presentation (%Δ day 1–15; Mean ± SEM).** Mean values of total time counted on day 1 for each behavioral category was used as baseline (black horizontal line). Grooming, sniffing and rearing behavior were summarized into one category (non-operant behavior) in order to make a clearer comparison between operant and non-operant behavior. *p < 0.05 (as compared with baseline); ^§^*p* < 0.05 (between groups).

Video analysis of behavior of the session times showed similar behavioral patterns on day 1 of the SRTT-training in lesion- and control-group: both groups showed no statistical difference in inactivity and non-operant behavior. However, the lesion-group spent less time with operant behavior (lesion: 546.96 ± 33.85 s; control: 685.63 ± 58.71 s; group difference: *F*_(1,17)_ = 4.745; *p* = 0.044). These patterns diverged over the course of training: descriptively, inactivity decreased in both groups, but only the lesion-group showed a statistical difference between day 1 and 15 (*F*_(1,20)_ = 16.673; *p* = 0.001). Operant behavior decreased in both groups and a statistical difference between day 1 and 15 was found (lesion: *F*_(1,20)_ = 16.046; *p* = 0.001; control: *F*_(1,14)_ = 7.488; *p* = 0.016). Non-operant behavior decreased only in the lesion-group (*F*_(1,20)_ = 7.477; *p* = 0.013), while the control-group showed no difference between day 1 and 15. A significant group difference in non-operant behavior on day 15 was found (*F*_(1,17)_ = 7.302; *p* = 0.015). Changes in time spent with each behavior from day 1 to day 15 (%Δ) are shown in Figure 4.

### Behavioral Results—Experiment 2

#### SRTT

**Figure 5:**
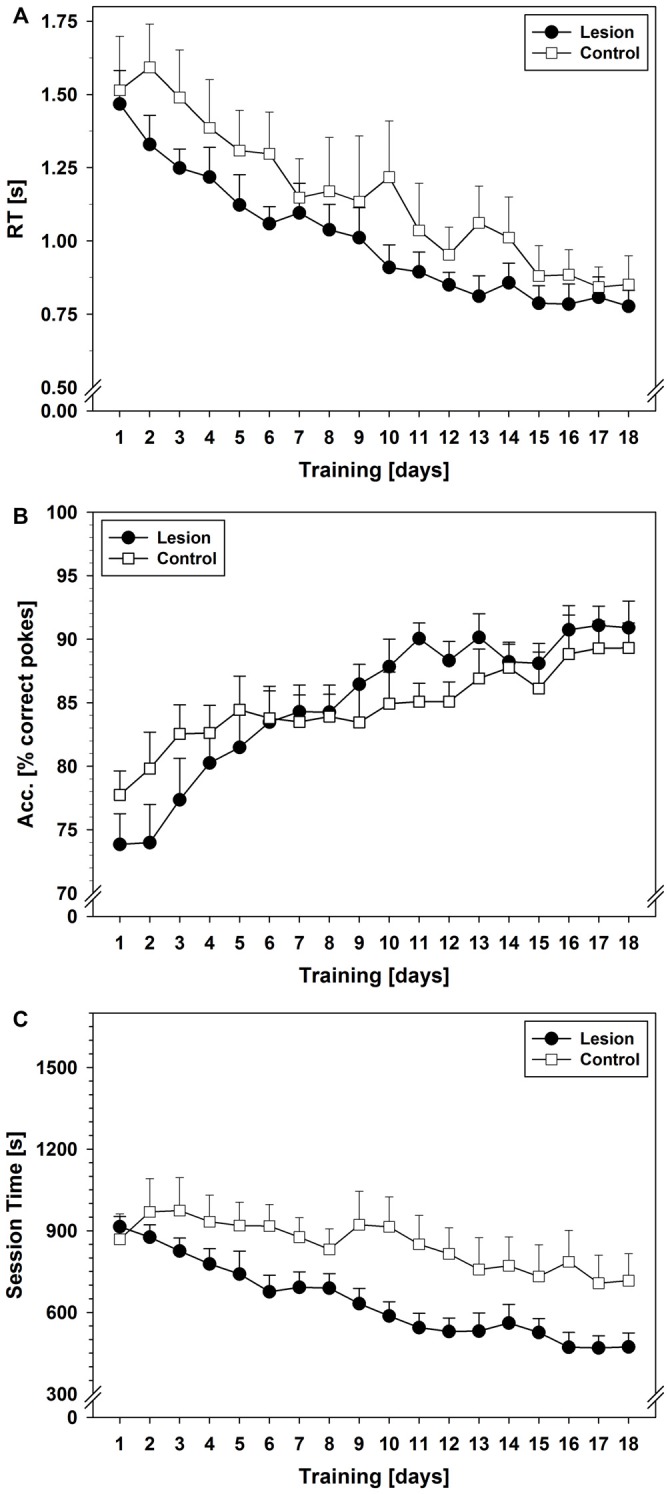
**SRTT data from Experiment 2. (A)** RTs in seconds during the 18-day training period. **(B)** Response ACC during the 18-day training period in terms of mean % of correct pokes. **(C)** Session times during the 18-day training period in seconds. Each data point reflects the mean of 20 successful FR-13 runs (±SEM). For statistical details see text.

Statistical analysis revealed that RTs of the correct pokes decreased over days and became asymptotic in both groups (factor days: *F*_(5.713,79.981)_ = 27.726; *p* < 0.001). The control-group showed slightly higher RTs at the beginning of the training period and greater inter-individual variability than the lesion-group (Figure 5A); however, there was no statistically significant group difference and no interaction between groups and days.

ACC increased during training in both groups (factor days: *F*_(4.911,68.761)_ = 13.801; *p* < 0.001). There was no significant difference between groups, but a trend for an interaction between groups and days (*F*_(4.911,68.761)_ = 2.112; *p* = 0.075). A stable level of performance was reached on day 16 in both groups (~90% correct pokes; Figure 5B).

Session time decreased in both groups over the training period (factor days: *F*_(3.954,55.357)_ = 13.352; *p* < 0.001). While the lesion-group showed a steady decrease in session time over 18 days, the control-group showed a much more shallow decrease (Figure 5C). Statistical analysis showed a significant group difference (*F*_(1,14)_ = 4.874; *p* = 0.044) and a trend for an interaction between the factor group and factor days (*F*_(3.954,55.357)_ = 2.359; *p* = 0.065).

**Figure 6:**
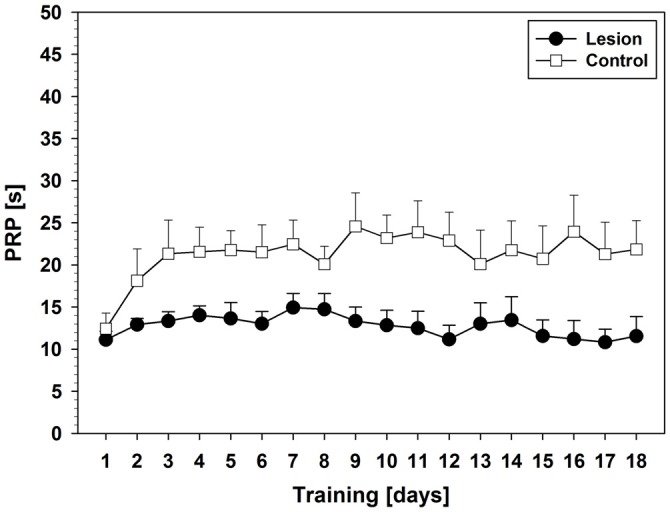
**PRPs from Experiment 2 (Mean ± SEM).** For statistical details see text.

Statistical analysis of PRPs revealed an effect on factor days (*F*_(17,238)_ = 2.097; *p* = 0.008) but only a trend in interaction between days and groups (*F*_(17,238)_ = 1.615; *p* = 0.061). Furthermore, a group difference was found, that is, shorter PRPs in the lesion-group (*F*_(1,14)_ = 8.589; *p* = 0.011). Unlike the lesion-group, the control-group showed an initial increase in PRPs (day 1–3). Thereafter, PRPs remained on a stable level throughout the rest of the training period (Figure 6).

#### Response-Outcome-Dissociation

**Figure 7:**
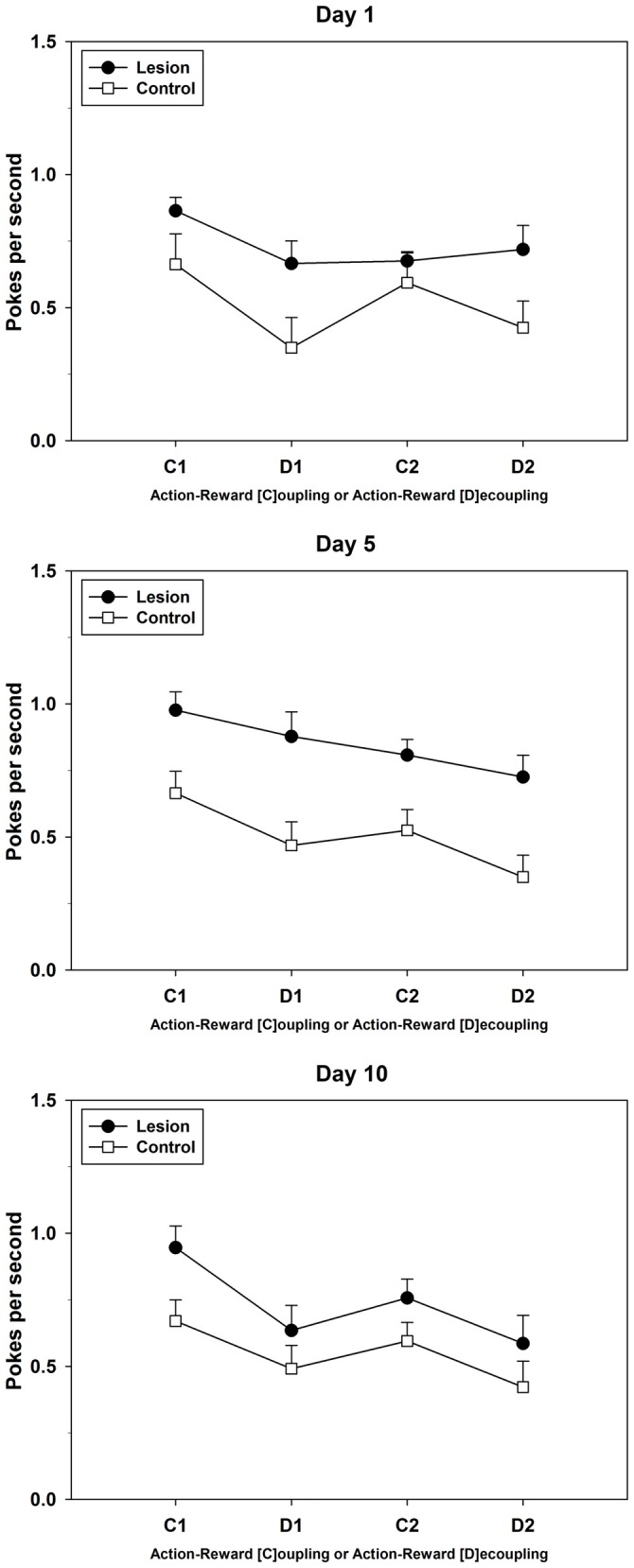
**Response-Outcome (R-O)-Dissociation.** Three exemplary (PPS)days of testing are shown (day 1, day 5, and day 10) with pokes per second during each of the four phases (Mean ± SEM) For statistical details see text.

On day 1 of this test, the control-group showed a saw-shape pattern of response, that is, fewer PPS during both R-O-decoupled phases (D1 and D2) as compared to those during the R-O-coupled phases (C1 and C2). The lesion-group showed fewer PPS during phase D1, but did not increase the amount of PPS in the subsequent C2-phase. From day 2 onwards until day 6 (data not shown in detail), the response pattern of the lesion-group resembled a sloping curve, with the greatest amount of PPS during the first phase, i.e., C1, and the least amount of PPS during the final phase, i.e., D2. In contrast, the response pattern of the control-group maintained the initially observed saw-shape. Starting on day 7, the lesion-group began to show signs of a saw-shape response pattern similar to the one observed in the control-group. Until the last day of testing, the response patterns of both groups aligned more and more and on day 10 the lesion-group showed a similar response pattern to the one presented by the control-group (see Figure 7 for three exemplary test days). Repeated measures ANOVAs for test days 1, 5 and 10, which were chosen for their evenly spaced chronological interval, yielded the following results: effects on factor phase were found on all 3 days (day 1: *F*_(3,42)_ = 15.556; *p* < 0.001; day 5: *F*_(3,42)_ = 17.168; *p* < 0.001; day 10: *F*_(1.993,27.900)_ = 14.745; *p* < 0.001), while an interaction between phases and groups was only found on day 1 (*F*_(3,42)_ = 3.090; *p* = 0.037). Group differences were only found on day 5 (*F*_(1,14)_ = 11.305; *p* = 0.005).

**Figure 8:**
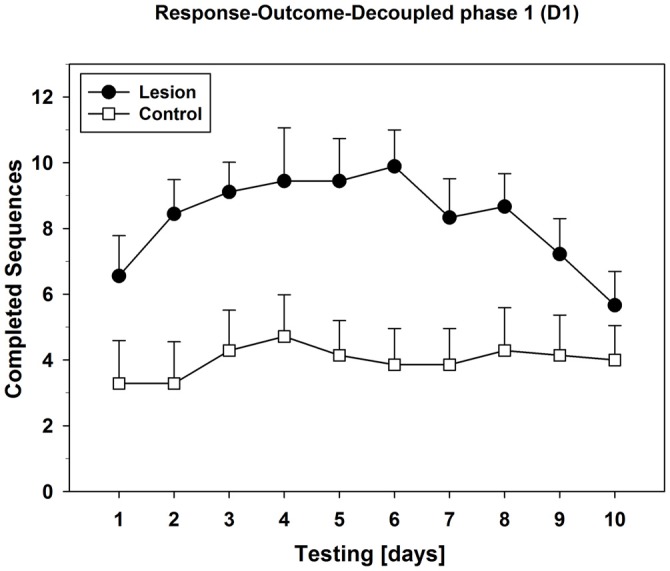
**Progression of the R-O-decoupled phase 1 (D1) over 10 days of testing.** Mean values (±SEM) of each D1-phase from all 10 days of testing were plotted subsequently in order to analyze behavioral progression of lesion- and control-group. For statistical details see text.

Also, we plotted the mean values of the daily completed sequences during phase D1 (Figure 8): The lesion-group showed a clear bell-curve-like progression over 10 days, while the control-group completed similar and lower amounts of sequences in phase D1 throughout the testing period. Specific analysis of phase-D1 with separate repeated measures ANOVAs for each group revealed a change over days in the lesion-group (*F*_(9,72)_ = 2.988; *p* = 0.004), but not in the control-group.

## Discussion

In this study, we provide evidence that dHC-ablated rats show enhanced instrumental performance under a FR-13 schedule with pseudorandom stimulus presentation. As in our prior study with sequential stimulus presentation (Busse and Schwarting, 2016), differences were found primarily in terms of shorter daily session times and shorter PRPs. In addition, a detailed video analysis showed that lesioned rats spent less time with operant behavior and simultaneously decreased non-operant behavior (grooming, sniffing, and rearing) over the course of the SRTT-training period. Furthermore, they showed impaired extinction behavior under devalued food reward conditions and, unlike controls, were apparently less able to discriminate between R-O-coupled and –decoupled task requirements.

The result of enhanced instrumental performance after dHC lesion appears counterintuitive at first sight, especially since deficits after complete or partial HC lesions are more common. These include deficits in spatial learning and memory (e.g., McDonald and White, 1994; Schroeder et al., 2002; Eckart et al., 2012; Busse and Schwarting, 2016), impairments in object recognition (Clarke et al., 2010), Pavlovian eyeblink conditioning (Gruart et al., 2006) and fear conditioning (McHugh and Tonegawa, 2009). However, findings on functional facilitation after HC lesions have also been reported, typically in avoidance tasks (Guillazo-Blanch et al., 2002; Wang et al., 2015), Pavlovian conditioning (Desmedt et al., 2003; Lee and Kim, 2004) and a variety of instrumental tasks (Gallagher and Holland, 1992; Compton, 2004; Cheung and Cardinal, 2005; for review, see Schwarting and Busse, submitted). Therefore, the consequences of HC lesions seem to be highly depended on the given task requirements.

### Histological Analysis

Hippocampal tissue reduction in the lesion-groups of both present experiments was similar to our previous studies where the same excitotoxic technique was used (Eckart et al., 2012; Busse and Schwarting, 2016), that is, the lesion led to a 40–60% loss of total hippocampal estimated volume, which mainly encompassed the dorsal HC and parts of the underlying intermediate HC, but spared the ventral part of the HC.

### Sham-Operated Animals

The sham-treated animals of Experiment 1 showed longer RTs, daily session times and PRPs as compared to untreated controls, even though gross tissue damage due to the sham procedure was small and not statistically significant compared to controls. These findings are in line with our previous data that consistently showed worse instrumental performance in rats with sham lesions. Possible explanations for such sham effects are multifaceted: sham surgical procedures are known to cause neurochemical and behavioral changes, including mnestic ones (Adams et al., 1994; Raghavendra Rao et al., 2000; Grossman et al., 2003; Hirshler et al., 2010). These may be due to various factors or their interactions, including anesthesia, skull surgery, cannula insertion, and saline injection. These may also have played a role in dHC-ablated animals, but performance facilitation by loss of hippocampal function may have overshadowed those effects. Sham-surgeries are generally very important in their function as a control, though in the context of our experiments the ambiguous behavioral changes only aggravated interpretation of our findings instead of providing a more clear understanding of lesion-induced facilitation of instrumental performance. Since we applied the exact same surgical procedures for both lesion- and sham-groups over the course of four distinct experiments (see Eckart et al., 2012; Busse and Schwarting, 2016), with the only difference being the injected substance (ibotenic acid vs. saline), it was not possible for us to determine why the sham-groups performed so poorly overall. Therefore, the results from Experiment 1 led us to the decision to forgo an additional sham-operated group in Experiment 2.

### SRTT

Results from the SRTT-training in both experiments were comparable and similar to our previous studies. All animals increased their response ACC towards ~90% correct pokes and decreased their RTs, as well as daily session times over the course of training. In Experiment 1, the lesion-group showed shorter RTs during the first days of training, but this difference vanished later around training day 10, at least in comparison to the control-group. In contrast, no such difference was found between control and lesioned rats in Experiment 2. This may be a result of the shaping phase that preceded the SRTT-training, where it is difficult to control for equal amounts of instrumental learning experience across all individual animals. Furthermore, the shaping phase in Experiment 1 lasted 7 days, while it took only 6 days to complete the shaping phase in Experiment 2. This one-day difference may have contributed to outcome differences between both experiments.

The lesion-group showed shorter daily session times in both experiments when compared to controls or sham-animals. This difference persisted throughout the training period. Interestingly, in Experiment 2, lesion- and control-group showed nearly identical daily session times on training day 1. Towards the end of the training period, however, these values had diverged significantly with the lesion-group reducing daily session times further than the control-group.

PRP data showed similar curve shapes in both experiments, with lesion animals maintaining their shorter PRPs throughout the whole training period. The control- and sham-rats initially increased PRP times two- or three-fold in comparison to lesion rats. They then remained stable on this level until the end of training. These differences likely play a major role in the group disparity observed in daily session times.

Since hyperactivity has been reported in rats after HC lesions (Andersen et al., 2007), it is possible that such a factor could have contributed to the observed differences in session time and PRPs. In our earlier studies we found mild signs (i.e., a statistical trend) for hyperactivity, namely increased locomotor activity during the habituation phase of an object place recognition task (Eckart et al., 2012), in dHC-ablated rats. However, the RTs do not support such a conclusion since lesion- and control-groups showed no significant statistical difference in RT during both present experiments.

### Pseudorandom vs. Sequential Stimulus Presentation

After switching from pseudorandom to sequential stimulus presentation on training day 17 in Experiment 1, we expected an increase in ACC and a decrease in RTs in all groups, and therefore, a decrease in daily session times. However, even though statistical significant improvements were found, the overall extent of a performance improvement by sequential stimulus presentation was surprisingly marginal. This was most likely the result of a ceiling effect, which may have prevented rats from improving their performance further. Since all animals reached their performance maximum after 16 days of training under this pseudorandom stimulus presentation, we adopted these findings in the design of our second experiment and used a pseudorandom stimulus presentation. This is comparable to the time span in our previous study where we used a sequential stimulus presentation (17 days of training until maximum performance). Overall, these results implied that the performance facilitation caused by dHC lesions is not specifically tied to sequential performance, but relates to an effect on instrumental performance in general.

### Devaluation-Extinction-Test

Under extinction conditions with a devalued food reward, the lesion-group of Experiment 1 completed more FR-13 runs and showed longer session times. Descriptively, these data implicate impaired extinction behavior in the dHC-group, but the statistical analysis revealed only a trend for a difference between lesion- and sham/control-groups. A statistical power analysis showed that both findings had only medium effect sizes (total time: effect size *f* = 0.529; total sequences: effect size *f* = 0.540) and therefore a power below 0.8, which could explain the statistical trend results. Nevertheless, the descriptive findings are in line with our previous study, where we found impaired extinction under non-devalued reward conditions in the dHC-group (Busse and Schwarting, 2016).

Both findings are in contrast to similar research from Corbit and Balleine (2000): they concluded in the first experiment of their study that dHC ablations had no effect on extinction behavior, since lesioned animals were as sensitive to instrumental outcome devaluation as sham-operated animals. However, it has to be noted that their procedures (fixed interval-20-schedule, two-day training period, two-choice task, electrolytic instead of excitotoxic lesions) differed substantially from the ones used in our study. Additionally, earlier studies have reported extinction impairments as a result of HC lesions in a variety of different learning tasks (avoidance tasks: Isaacson et al., 1961; Green et al., 1967; Tonkiss et al., 1990; Weiner et al., 1998; Guillazo-Blanch et al., 2002; Pavlovian conditioning: Klüver, 1965; Schmaltz and Theios, 1972; instrumental tasks: Clark and Isaacson, 1965; Schmaltz and Isaacson, 1966; Brown et al., 1969; for review, see also Schwarting and Busse, submitted). It has also previously been shown that extensive instrumental training can lead to behavior that is initially goal-directed and mainly dependent upon the dorsomedial striatum (DMS) and then becomes habitual and largely dependent upon the dorsolateral striatum (DLS; Graybiel, 2008). Prolonged SRTT-training as it was used in our study most certainly led to a shift from R-O towards S-R behavior. The dHC lesions may have facilitated this process due to a change of direct or indirect HC interaction with the DMS and DLS. This could explain why an extinction impairment was present in our lesion-group, but not in the study from Corbit and Balleine (2000). Consequentially, the occurrence of impaired extinction behavior as a result of dHC lesions seems to be highly dependent on the given learning task and the amount of training that is provided.

Finally, we should address the question which factors might have determined extinction in our test, since extinction was not only characterized by the lack of reward, but also stimuli usually paralleling its delivery, namely sounds made by the food dispenser and the dropping food pellets. Thus, the differences between control and dHC-ablated rats may be due to reward omission, discontinuation of discriminative stimuli, or interactions between both. This limitation must be considered when interpreting these results.

### Behavioral Recordings

Analysis of behavioral observations in Experiment 1 revealed another layer of the complex changes resulting from dHC ablations: while time spent with operant behavior during the session decreased in lesion- and control-group to a similar degree, non-operant behaviors (grooming, sniffing, and rearing) disappeared almost entirely in dHC-ablated rats. In contrast, intact rats pursued non-operant behaviors even after prolonged training, with no changes seen in the time spent from the first day of training in comparison to subsequent days. Differences seen in non-operant behavior contributed substantially to the divergence in daily session times between dHC-ablated and intact animals. It can be assumed that the reductions in non-operant behavior, as well as the shorter PRPs, constituted the main behavioral alterations that led to the instrumental performance increase in the lesion-group. However, it is important to note that this performance increase may be only marginally, or not at all, related to actual strengthening of the instrumental learning process, but rather, to a state in which the dHC-ablated animals apparently had no other motivation than completing the instrumental task. Therefore, they exceeded controls and shams in performance. Such a motivational change may underlie several behavioral alterations in rats with hippocampal lesions: reports of decreased awareness for potentially aversive or distracting stimuli (Chudasama et al., 2008, 2009; Machado and Bachevalier, 2008), as well as diminished behavior driven by motivational states and internal needs, e.g., lack of frustration and “hesitation and doubt”, can be found throughout the hippocampal literature (Kimble, 1968; Coover et al., 1971; Isaacson and Kimble, 1972; Hirsh, 1974; Simonov, 1974, 1991; see also Schwarting and Busse, submitted).

### Response-Outcome-Dissociation

In this test of Experiment 2, control-animals showed a clear ability to discriminate between the phases where rewards were dependent on their actions vs. those where they were not, since they decreased their nose-pokes during the decoupled phases leading to a saw-shape response pattern (Figure 7). This effect was observed starting from day 1 and lasted until the end of this testing period. In contrast, dHC-ablated rats did not only show higher rates of PPS but were apparently able to differentiate less between coupled and decoupled task phases. Similar patterns were observed on test day 5, whereas on test day 10, the lesion-group also showed decreased responding during the decoupled phases. Therefore, the dHC-lesioned animals displayed an initial impairment in the ability of correctly assessing the task requirements of each phase, while the control-group responded correctly right from the start. Further analyses of completed sequences during the first decoupled phase revealed that dHC-ablated rats showed a bell-curve-like progression of performance over 10 days of testing (Figure 8), which indicates S-R behavior up until test day 6. Since their initial behavior could be interpreted as indecisive on which instrumental strategy was best for the present task, the dHC-ablated rats displayed a clear habitual- instead of a goal-directed strategy after test day 2. This made them operate similarly during both R-O-task conditions and it resulted in higher PPS, as well as more completed sequences during the decoupled phases than the control-group. These findings indicate that the ability of correctly assessing task requirements was not lost completely, but it took much longer for the dHC-lesioned rats to use it and adapt their behavioral strategy, possibly because their behavior was largely determined by S-R rather than R-O mechanisms.

A similar effect on R-O-relations has been shown in the second experiment conducted in the study by Corbit and Balleine (2000), where intact animals pushed a lever less frequently for which the previously associated action-outcome contingency had been degraded, as opposed to a second lever for which the previously established action-outcome contingency had been preserved. In comparison, rats with dHC lesions responded to both levers at equal rates. They concluded that this supports a specific interpretation of the role of the HC in declarative memory which states that the hippocampal formation is integral for the detection of the causal relationships between actions and outcomes (Squire and Zola, 1996; Wise and Murray, 2000) and that damage to the HC renders rats unable to differentiate between “actions that are causal with respect to their associated outcomes and those that are merely adventitiously related” (Corbit and Balleine, 2000).

Several possible interpretations can be drawn from the present results: firstly, the dHC may have direct or indirect influence on processes within the DMS or DLS, which are known to play a major role in the realization of R-O or S-R instrumental contingencies and strategies. Several studies have shown that the DMS is involved in R-O learning and prolonged behavioral training results in a transfer of behavioral control from R-O associations to S-R habit systems, which is believed to be mediated by the DLS (Adams and Dickinson, 1981; Yin et al., 2004, 2005; Daw et al., 2005; Yin and Knowlton, 2006). Ablation of dHC structures might disrupt these processes and result in a preferential or faster establishment of S-R behavior than in intact animals. Simultaneously, if hippocampal interaction with the DMS is, at least in part, a prerequisite for R-O associations, a reversal towards goal-directed behavior could be aggravated and would rely on prolonged exposure to the changed environment, i.e., different task conditions. In more general terms, the influence of the dHC could possibly inhibit or slow down transitions from goal-directed to habitual behavior under normal conditions. However, anatomical and behavioral data that support this idea are inconclusive: the DMS receives a wide array of inputs from different parts of the brain, e.g., prefrontal cortices, entorhinal cortex, subiculum, hippocampus, amygdala, thalamus, and piriform cortex (McGeorge and Faull, 1989; Voorn et al., 2004; Gruber and McDonald, 2012). Nonetheless, interplay between DMS and the hippocampal formation seems to be of particular interest since evidence shows that direct and indirect neuronal connections between both areas are rather complex and multi-layered. Studies by Krayniak et al. (1981) and Swanson and Köhler (1986) reported extensive projections from the entorhinal cortex, *inter alia*, into the DMS and nucleus accumbens. Furthermore, Groenewegen et al. (1987) were able to show that the subiculum, as the main output structure of the hippocampal formation, projects into many parts of the medial and ventral striatum. Expanding on the findings of McGeorge and Faull (1989) showed that the CA1 field of the HC also directly projects into the nucleus accumbens. Finally, projections from the dHC towards the posterior cingulate cortex, which itself projects strongly to the DMS, and projections from the ventral HC towards the medial prefrontal cortex, which then again projects into the DMS, have been reported by McGeorge and Faull (1989) in the same study.

In contrast, functional studies yielded ambiguous and nonspecific findings regarding interactions of the hippocampal formation and indirect sources of hippocampal input with the DMS and also other striatal regions (e.g., ventral striatum/nucleus accumbens). Lesions of brain regions that pass through inputs from the hippocampal formation to the DMS result in impairments in place learning, when rats are tested in the water maze task (Schenk and Morris, 1985; Sutherland et al., 1988; Kolb et al., 1994; Ferbinteanu et al., 1999), which makes it difficult to assess if any region of the hippocampal formation has preferential influence on the DMS. Additionally, DMS lesions also resulted in impairments in place learning, when rats were tested in a variant of this task, in which they had to adapt their behavior to a submerged platform that changed its position after every 8th trial. However, in contrast to dorsal or ventral HC-lesioned rats, animals with a DMS lesion still showed within-session improvements during this task (Ferbinteanu et al., 2003; McDonald et al., 2008). These data indicate that the DMS may play a role in response flexibility after animals are confronted with changes in the environment, especially regarding tasks in which spatial navigation is required, e.g., spatial reversal learning tasks (Castañé et al., 2010).

Secondly, loss of hippocampal function and therefore lesser impacts on other brain areas, e.g., prefrontal cortices, may strengthen pathways between DMS or DLS and prefrontal structures. This could have fastened transition into habitual behavior, caused by less competition or interference between declarative and non-declarative memory systems. This interpretation is supported by several studies and reviews that comprehensively analyzed behavioral changes after micro-infusions into, or lesioning of different brain structures that are deemed part of the “multiple parallel memory systems” hypothesis (Mink, 1996; Packard, 1999; White and McDonald, 2002; McDonald et al., 2007; Gruber and McDonald, 2012).

Thirdly, damage to dorsal portions of the HC may have resulted in functional changes within the remaining areas of the hippocampal formation, i.e., ventral and intermediate HC. The hippocampal formation displays strong intrahippocampal connections (Amaral and Witter, 1989), which in return suggests mutual modulation of hippocampal sub-regions. It is firmly established that spatial learning and memory is more related to dHC structures, while modulation of sensorimotor processes is more related to ventral HC structures (Bast and Feldon, 2003; Chen et al., 2010; McHugh et al., 2011), but these anatomical and functional differences are expressed as a gradient along the septo-temporal axis and cannot be seen as distinct structures within the hippocampal formation. Furthermore, it has been shown that the intermediate HC is able to functionally compensate damage to dorsal and ventral HC structures (Bast et al., 2009; Bast, 2011). Although the intermediate portion of the hippocampal formation was still mainly intact in our lesioned animals, the extensive damage to the dHC may have resulted in alterations of downstream processing towards the remaining HC and striatal structures, which in return led to the observed behavioral changes.

It is important to note that none of these interpretations are exclusive and more comprehensive research regarding behavioral alterations after damage to, or ablations of adjacent brain areas not directly associated with a given memory system, is necessary in order to provide further understanding of interactions between them. However, all of this evidence combined suggests that sensory input processing within the hippocampal formation may be closely linked with translation and contextualization of sensory information, towards and within the striatum and finally, with the resulting behaviors.

## Conclusion

The data obtained in this study provide further evidence for an enhancing effect of dHC lesions on instrumental performance, that is, the previously found deficits in sequential behavior, were due to a more general deficit affecting instrumental behavior, rather than a specific sequential deficit. Still, the question of why and how damage to the hippocampal formation may result in beneficial behavioral changes in instrumental tasks needs to be raised, as it is functionally associated with declarative memory systems, whereas instrumental tasks, which are typically associated with non-declarative memory systems, are functionally tied to an anatomically distinct brain structure, i.e., the striatum. In our example, lesion of the dHC apparently led to facilitation of the behavioral shift from goal-directed to habitual behavior, which turned out to be a more effective strategy for the dHC-ablated rats in the present instrumental learning task. In return, dHC-ablated rats were impaired in the ability to adapt to a sudden change in the environment, i.e., the rules on how and when to obtain a food reward. Therefore, it took them much longer to reverse habitual back to goal-directed behavior. This suggests that information processing within the dHC may signal changes in the environment. By this means it can then take part in the decision on which instrumental behaviors should remain goal-directed and which can become habitual. Without it, instrumental behaviors may be prone to become habitual regardless of the environmental contingencies.

## Author Contributions

SB and RKWS designed the study; SB performed surgery and behavioral experiments, RKWS supervised experiments; SB collected and analyzed data, RKWS supervised analysis; Both authors interpreted the data and discussed the results; SB wrote the manuscript, RKWS edited the manuscript.

## Funding

This work was supported by grant SCHW 559/12-1 from the Deutsche Forschungsgemeinschaft.

## Conflict of Interest Statement

The authors declare that the research was conducted in the absence of any commercial or financial relationships that could be construed as a potential conflict of interest.
